# Crystal structure and Hirshfeld surface analysis of a conformationally unsymmetrical bis­chalcone: (1*E*,4*E*)-1,5-bis­(4-bromo­phen­yl)penta-1,4-dien-3-one

**DOI:** 10.1107/S2056989019006480

**Published:** 2019-05-10

**Authors:** Nabeel Arif Tawfeeq, Huey Chong Kwong, Mohamed Ibrahim Mohamed Tahir, Thahira B. S. A. Ravoof

**Affiliations:** a Department of Chemistry, Faculty of Science, Universiti Putra Malaysia, 43400 UPM Serdang, Selangor Darul Ehsan, Malaysia; b Department of Chemistry, College of Education for Women, University of Anbar, Iraq

**Keywords:** crystal structure, bis­chalcone, penta­dienone bridge, C—H⋯π inter­actions, Hirshfeld surface analysis

## Abstract

In the title bis­chalcone, C_17_H_12_Br_2_O, the olefinic double bonds are almost coplanar with their attached 4-bromo­phenyl rings [torsion angles = −10.2 (4) and −6.2 (4)°], while the carbonyl double bond is in an *s-trans* conformation with with respect to one of the C=C bonds and an *s-cis* conformation with respect to the other [C=C—C=O = 160.7 (3) and −15.2 (4)°, respectively]. The dihedral angle between the 4-bromo­phenyl rings is 51.56 (2)°. In the crystal, mol­ecules are linked into a zigzag chain propagating along [001] by weak C—H⋯π inter­actions. The conformations of related bis­chalcones are surveyed and a Hirshfeld surface analysis is used to investigate and qu­antify the inter­molecular contacts.

## Abstract 2

The title bis­chalcone shows *s-trans* and *s-cis* conformations for its C=C—C=O bonds.

## Chemical context   

Dibenzalacetone, or bis­chalcone, [(1*E*,4*E*)-1,5-di­phenyl­penta-1,4-dien-3-one] was first prepared by the base-catalyzed Aldol condensation of benzaldehyde and acetone (Conard & Morris, 1932 ▸): it results in a highly conjugated system involving the *α*,*β*-unsaturated penta­dienone (–C=C—(C=O)—C=C—) moiety. Bischalcones have a number of uses including anti-inflammatory (Mahapatra *et al.*, 2017 ▸) and anti-oxidant (Pandey & Syed, 2009 ▸) agents. Different bis­chalcones consist of two benzene rings substituted with different types of functional groups (electron donor or acceptor) bonded to the ends of the central *α*,*β*-unsaturated ketone which provides good configuration for the transfer of intra­molecular charge (Fun *et al.*, 2011 ▸). In a continuation of our ongoing studies on the non-linear optical properties of various chalcone derivatives (Sim *et al.*, 2017 ▸; Kwong *et al.*, 2018 ▸), we report herein the synthesis, structure determination and Hirshfeld surface analysis of the title compound (I).
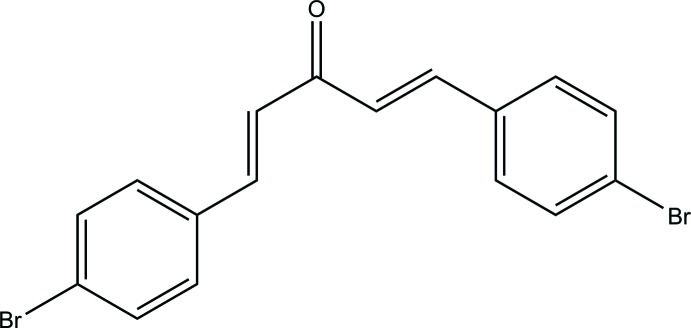


## Structural commentary   

**Figure 1:**
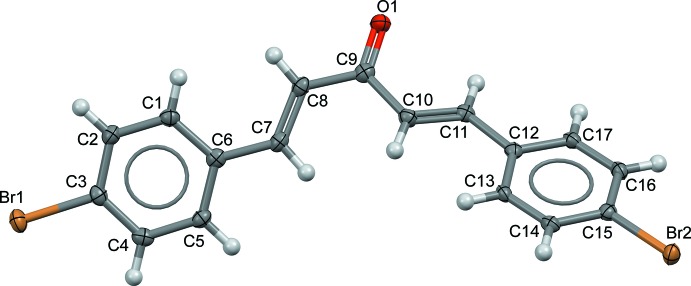
The mol­ecular structure of the title compound showing 50% displacement ellipsoids.

**Figure 2:**
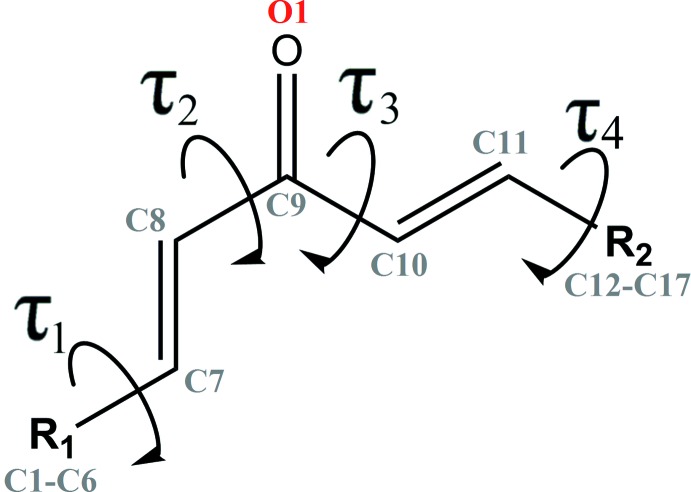
General chemical diagram showing torsion angles, *τ*_1_, *τ*_2_, *τ*_3_ and *τ*_4_ in the title compound.

The asymmetric unit of (I) consists of a single mol­ecule, consisting of two 4-bromo­phenyl rings connected by a penta-1,4-dien-3-one bridge (Fig. 1 ▸). The bond lengths and angles of the central chain are consistent with those in related structures (Butcher *et al.*, 2007*a* ▸; Ruanwas *et al.*, 2011 ▸). The overall conformation of (I) can be described by the the torsion angles between the olefinic double bonds and 4-bromo­phenyl rings [*τ*1 (C1—C6—C7—C8); *τ*4 (C13—C12—C11—C10)] and the carbonyl double bond [*τ*2 (C7—C8—C9—O1); *τ*3 (C11—C10—C9—O1)] (Fig. 2 ▸). The 4-bromo­phenyl rings in (I) are close to coplanar with their attached olefinic double bonds [*τ*_1_ = −10.2 (4)°; *τ*_4_ = −6.2 (4)°] but the conformations of the olefinic double bonds are very different: one is in *s-trans* [*τ*_2_ = 160.7 (3)°] conformation and in *s-cis* [*τ*_3_ = −15.2 (4)°] conformation with the central C=O double bond. These torsions result in an overall twisted shape for (I) with the dihedral angle between the 4-bromo­phenyl ring being 51.56 (2)°.

## Supra­molecular features   

**Figure 3:**
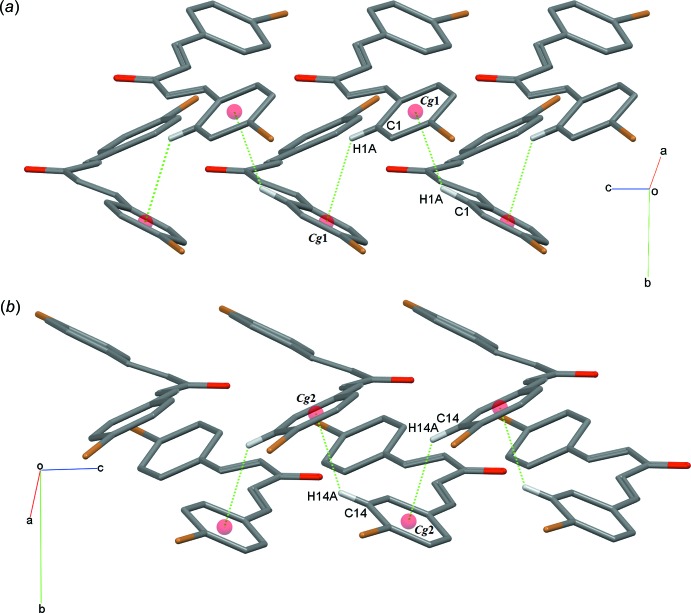
A partial packing diagram of the title compound, with (*a*) C1—H1*A*⋯π and (*b*) C14—H14*A*⋯π inter­actions (dotted lines). Hydrogen atoms not involved in these inter­actions have been omitted for clarity.

No classical hydrogen bonding is possible in (I) and in the crystal, mol­ecules are linked by C—H⋯π inter­actions (Table 1 ▸): the first of these results in a phen­yl–phenyl T-shaped geometry *via* C1—H1*A*⋯*Cg*1^i^ (Fig. 3 ▸ *a*). The C14—H14*A*⋯*Cg*2^ii^ (Fig. 3 ▸ *b*) inter­actions lead to a zigzag chain along the *c*-axis direction.

## Database survey   

A survey of the Cambridge Structural Database (CSD, version 5.40, last update February 2019; (Groom *et al.*, 2016 ▸)) using (1*E*,4*E*)-1,5-di­phenyl­penta-1,4-dien-3-one as the main skeleton revealed the presence of 27 structures containing a similar bis­chalcone moiety to the title compound but with different substituents on the terminal phenyl rings. The different substituents (**R_1_** and **R_2_**) together with the torsion angles of the penta-4,4-dien-3-one connecting bridge are compiled in Table 2 ▸. For the conformationally symmetrical compounds (i.e. both C=C—C=O bonds are either *s*-*cis* or *s*-*trans*), the olefinic double bonds are close to coplanar with their attached phenyl rings as indicated by their *τ*_1_ and *τ*_4_ torsion angles, which fall in the range of 0.0–17.8°, except for the compounds AMEXUN and HUDLEY, which have somewhat larger *τ*_1_ and *τ*_4_ values of 22.5–27.4°. The olefinic double bonds for the symmetrical compounds are mostly in *s-cis* conformations with the carbonyl double bond (*τ*_2_/*τ*_3_ torsion angles of 0.1–21.9°). However, both the olefinic double bonds of compounds GOLGOD and GOLGOD02 are in *s-trans* conformations with the carbonyl double bond (*τ*_2_/*τ*_3_ = 152.2–153.4°). Furthermore, it may be noted that the symmetrical conformation at the penta-4,4-dien-3-one connection bridge is not affected by the different substituents at the **R_1_** and **R_2_** positions in EDUSEE, SAFZOO and XOHVUN. Most of the unsymmetrical compounds (one C=C—C=O bond *s-cis* and one *s-trans*) have *τ*_1_ and *τ*_4_ values of 0.5–17.2°, which indicates that the olefinic double bonds are close to coplanar to their attached phenyl ring. The outliers are MESXEQ and WIHBUL, which have *τ*_1_ and *τ*_4_ values of 18.2–51.8° and 21.4–51.8°, respectively. The torsion angles *τ*_2_ and *τ*_3_ for the unsymmetrical compounds, including (I), are in the ranges 160.2–178.7° and 0.5–23.7°, respectively, which indicate *s-trans* and *s-cis* conformations between the olefinic double bonds and the carbonyl double bond.

## Hirshfeld surface analysis   

**Figure 4:**
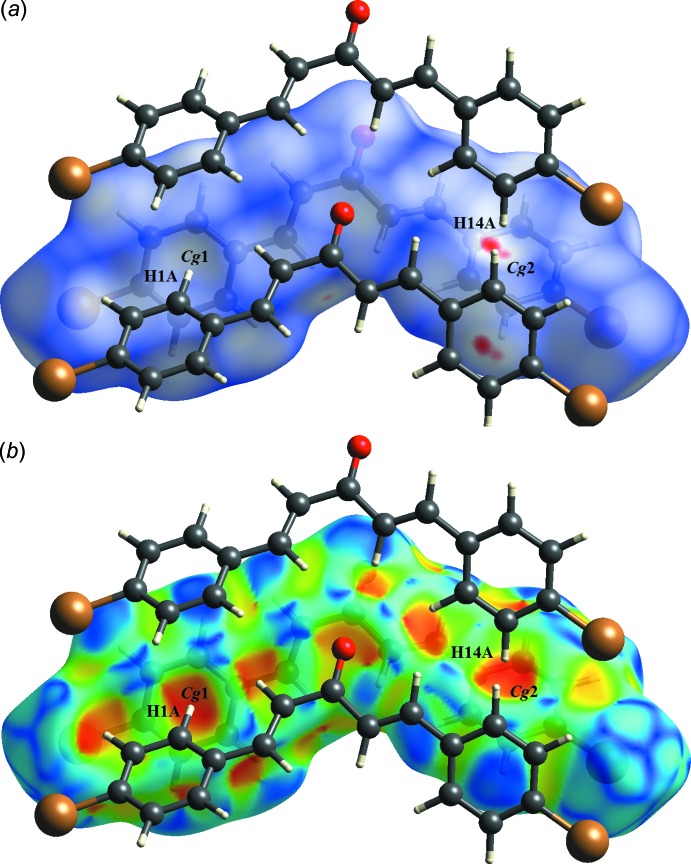
The Hirshfeld surface mapped with (*a*) *d*_norm_ and (*b*) shape-index for the title compound showing the C—H⋯π inter­actions.

**Figure 5:**
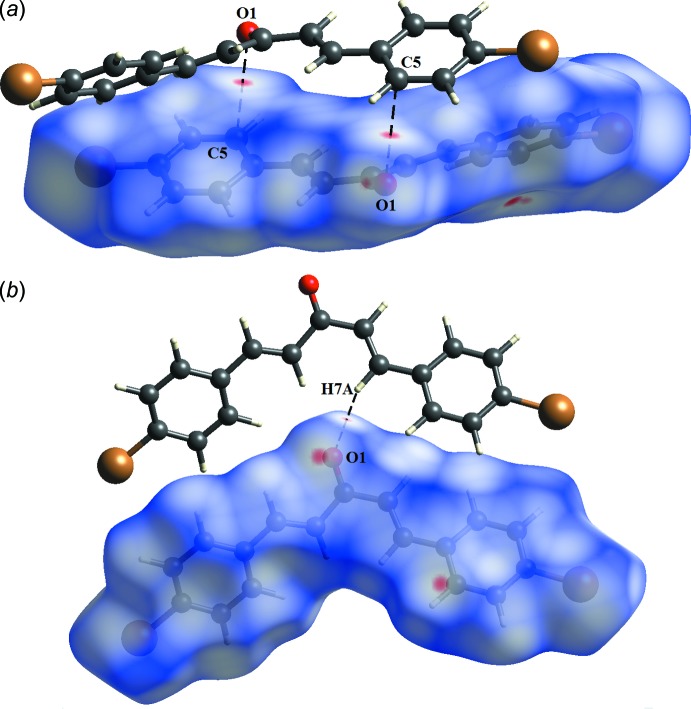
The Hirshfeld surface mapped with *d*_norm_ showing (*a*) the C5⋯O1 short contact and (*b*) the weak C7—H7*A*⋯O1 inter­action.

The Hirshfeld surfaces mapped with normalized contact distance *d*_norm_ and the two-dimensional fingerprint plots for (I) were generated using *CrystalExplorer17.5* (Turner *et al.*, 2017 ▸). The darkest red spots on the Hirshfeld surface mapped with *d*_norm_ (Fig. 4 ▸ *a*) correspond to the C14—H14*A*⋯*Cg*2^ii^ inter­action. Even through the C1—H1*A*⋯*Cg*1^i^ inter­action is not visible in the *d*_norm_ surface mapping, this inter­action can be seen as a unique pattern of a red ‘circle’ on the shape-index surface mapping (Fig. 4 ▸ *b*). Besides the C—H⋯π inter­actions, the *d*_norm_ surface mapping indicated a short contact between atom O1 and C5 with a distance of 0.06 Å shorter than the sum of the van der Waals radii of O and C atoms (3.22 Å; Fig. 5 ▸ *a*). Together with this short contact, another weak C7—H7*A*⋯O1 inter­action was also revealed as light spots on the *d*_norm_ surface (Fig. 5 ▸ *b*).

**Figure 6:**
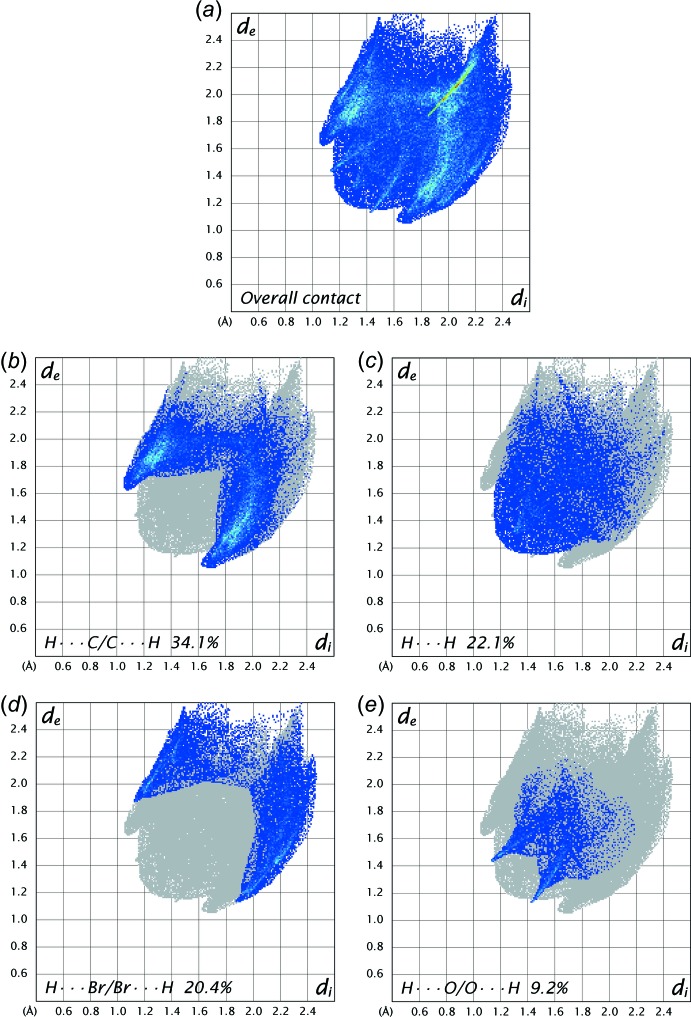
The two-dimensional fingerprint plots of the title compound for different inter­molecular contacts and their percentage contributions to the Hirshfeld surface. *d*_e_ and *d*_i_ are the distances from the Hirshfeld surface to the nearest atom inter­ior and exterior, respectively, to the surface.

As illustrated in Fig. 6 ▸, the corresponding fingerprint plots for (I) are shown with characteristic pseudo-symmetric wings in the *d*_e_ and *d*_i_ diagonal axes. The H⋯C/C⋯H contacts are the most populated contacts and contribute 34.1% to the total inter­molecular contacts, followed by H⋯H (22.1%), H⋯Br/Br⋯H (20.4%) and H⋯O/O⋯H (9.2%) contacts (Fig. 6 ▸). As the C—H⋯π bonds are the main inter­action in the crystal, the most populated H⋯C/C⋯H contacts appear as two symmetrical narrow wings at diagonal axes *d*_e_ + *d*_i_ ≃ 2.7 Å (Fig. 6 ▸ *b*). The H⋯H contacts appear in the central region of the fingerprint plots with *d*_e_ = *d*_i_ = 2.4 Å (Fig. 6 ▸ *c*). With the presence of relatively larger bromine atoms in the structure, the H⋯Br/Br⋯H contacts appear as symmetrical broad wing at diagonal axes of *d*_e_ + *d*_i_ ≃ 3.0 Å (Fig. 6 ▸ *d*). Two symmetric spikes in the fingerprint plots with a short spike at *d*_e_ + *d*_i_ ≃ 2.7 Å represent the H⋯O/O⋯H contacts (Fig. 6 ▸ *e*), indicating the presence of the weak C7—H7*A*⋯O1 inter­action. The percentage contributions for other contacts are less than 15% in the Hirshfeld surface mapping.

## Synthesis and crystallization   

A mixture of 4-bromo­benzaldehyde (4.9 g, 12.5 mmol) and acetone (0.363 g, 6.25 mmol) dissolved in absolute ethanol (30 ml) was slowly added to an aqueous solution of potassium hydroxide (4.0 g in 20 ml water). The mixture was vigorously stirred at room temperature for two h and then 20 ml chilled water was added. The resulting yellow precipitate was recovered by vacuum filtration and washed with cold water (100 ml). The crude product was recrystallized from absolute ethanol solution as yellow blocks.

***(1E,4E)-1,5-Bis­(4-bromo­phen­yl)penta-1,4-dien-3-one***; pure yellow solid (4.6 g, 88.6%), m.p. 484 K; **IR** ν_max_ 594, 687, 813, 979, 1066, 1181, 1320, 1398, 1480, 1581, 1643 cm^−1^, **UV–Vis** λ_max_. 227 and 317 nm, **^1^H NMR:** *δ*_H_ (500MHz, CDCl_3_) 7.02 (2H, H-1), 7.45 (4H, H-2), 7.54 (4H, H-3), 7.67 (2H, H-4); **^13^C NMR:** *δ*_C_ (125MHz, CDCl_3_) 124.62, 125.69, 129.25, 131.74, 133.29, 141.83, 188.22; **HRMS** (ES): *M*H^+^, found: 392 C_17_H_12_Br_2_O^+^ requires: 391.92.

## Refinement   

Crystal data, data collection and structure refinement details are summarized in Table 3 ▸. C-bound H atoms were positioned geometrically (C–H = 0.93 Å) and refined using a riding model with *U*_iso_(H) = 1.5*U*_eq_(C).

## Supplementary Material

Crystal structure: contains datablock(s) I. DOI: 10.1107/S2056989019006480/hb7821sup1.cif

Structure factors: contains datablock(s) I. DOI: 10.1107/S2056989019006480/hb7821Isup2.hkl

Supporting information file. DOI: 10.1107/S2056989019006480/hb7821Isup3.cml

CCDC reference: 1914420

Additional supporting information:  crystallographic information; 3D view; checkCIF report

## Figures and Tables

**Table 1:** Hydrogen-bond geometry (Å, °) *Cg*1 and *Cg*2 are the centroids of the C1–C16 and C12–C17 rings, respectively.

*D*—H⋯*A*	*D*—H	H⋯*A*	*D*⋯*A*	*D*—H⋯*A*
C1—H1*A*⋯*Cg*1^i^	0.93	2.86	3.539 (3)	131
C14—H14*A*⋯*Cg*2^ii^	0.93	2.74	3.428 (3)	131

Symmetry codes: (i) 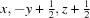; (ii) 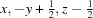.

**Table 2:** Torsion angles τ_1_, τ_2_, τ_3_ and τ_4_ (°)

Compound	**R_1_**	**R_2_**	τ_1_	τ_2_	τ_3_	τ_4_
Symmetrical						
AMEXUN (Mark *et al.*, 2016 ▸)	4-(benz­yloxy)-3-meth­oxy­phen­yl	4-(benz­yloxy)-3-meth­oxy­phen­yl	27.4	19.3	20.4	22.5
COGNOD01 (Rawal *et al.*, 2016 ▸)	4-(di­ethyl­amino)­phen­yl	4-(di­ethyl­amino)­phen­yl	1.2, 4.0	11.0, 4.2	1.3, 2.9	0.7, 1.6
DUMWIS (Fun *et al.*, 2010 ▸)	2,4,5-tri­meth­oxy­phen­yl	2,4,5-tri­meth­oxy­phen­yl	11.6, 0.4, 11.8	0.7, 4.0, 5.5	3.0, 8.2, 6.0	9.8, 2.9, 3.7
EDUSEE (Rawal *et al.*, 2016 ▸)	4-(di­ethyl­amino)­phen­yl	4-benzo­nitrile	15.6, 5.9	0.1, 6.4	8.1, 7.7	3.7, 3.2
GOLGOD (Shan *et al.*, 1999 ▸)	4-meth­oxy­phen­yl	4-meth­oxy­phen­yl	3.2	153.4	152.9	2.7
GOLGOD02 (Harrison *et al.*, 2006 ▸)	4-meth­oxy­phen­yl	4-meth­oxy­phen­yl	2.4	152.2	152.2	2.4
HIDMIQ (Zhou *et al.*, 1999 ▸)	2-meth­oxy­phen­yl	2-meth­oxy­phen­yl	0.2	1.4	1.2	8.8
HUDLEY (Feng *et al.*, 2009 ▸)	2,4-di­methyl­pheny	2,4-di­methyl­pheny	26.1	3.1	1.1	24.6
KOFCEO (Arshad *et al.*, 2008 ▸)	4-methyl­phen­yl	4-methyl­phen­yl	16.6	4.0	13	180
LEJNOE (Butcher *et al.*, 2006 ▸)	4-chloro­phen­yl	4-chloro­phen­yl	17.8	9.8	9.8	17.8
LESGAT (Park *et al.*, 2013 ▸)	2-(tri­fluoro­meth­yl)phen­yl	2-(tri­fluoro­meth­yl)phen­yl	0.5	0.3	2.9	13.7
SAFZOQ (Samshuddin *et al.*, 2012 ▸)	3-nitro­pheny	phen­yl	6.1	11.3	21.9	10.4
SIMTUE (Nizam Mohideen *et al.*, 2007 ▸)	2-chloro­phen­yl	2-chloro­phen­yl	8.8	3.4	0.9	0.5
UPAWEO (Huang *et al.*, 2011 ▸)	2,6-di­fluoro­phen­yl	2,6-di­fluoro­phen­yl	2.3	4.4	0.8	178
UPAWEO01 (Schwarzer & Weber, 2014*a* ▸)	2,6-di­fluoro­phen­yl	2,6-di­fluoro­phen­yl	2.3	4.4	0.8	0.5
WACXON (Hubig *et al.*, 1992 ▸)	*o*-tol­yl	*o*-tol­yl	10.3	1.1	2.8	1.5
XOHVOH (Schwarzer & Weber, 2014*b* ▸)	penta­fluoro­phen­yl	penta­fluoro­phen­yl	3.0, 7.9	1.0, 5.7	1.6, 3.4	5.7, 2.3
XOHVUN (Schwarzer & Weber, 2014*b* ▸)	penta­fluoro­phen­yl	phen­yl	5.6	2.4	3.3	7.8
						
Unsymmetrical						
(I)	4-bromo­phen­yl	4-bromo­phen­yl	10.2	160.7	15.2	6.2
IFAQAJ (Kapdi *et al.*, 2013 ▸)	3,5-di­meth­oxy­phen­yl	3,5-di­meth­oxy­phen­yl	5.4	173.1	0.6	3.2
LEJNOE01 (Maluleka & Mphahlele, 2017 ▸)	4-chloro­phen­yl	4-chloro­phen­yl	11.2	160.2	13.6	6.6
MESXEQ (Dravida *et al.*, 2018 ▸)	2,6-di­chloro­phen­yl	2,6-di­chloro­phen­yl	46.8, 48.7, 51.8	175.3, 4.3, 178.7	7.5, 4.3, 15.5	32.7, 48.7, 51.8
QAJNOG (Ruanwas *et al.*, 2011 ▸)	2,4,6-tri­meth­oxy­phen­yl	2,4,6-tri­meth­oxy­phen­yl	6.6, 0.5	176.8, 169.4	1.2, 0.5	3.7, 11.8
WIHBUL (Butcher *et al.*, 2007*a* ▸)	4-fluoro­phen­yl	4-fluoro­phen­yl	18.2, 18.7	169.0, 166.3	10.4, 8.8	21.8, 21.4
XIFTOW (Butcher *et al.*, 2007*b* ▸)	3,4-di­meth­oxy­phen­yl	3,4-di­meth­oxy­phen­yl	1.6, 1.6	162.8, 170.8	23.3, 23.7	3.1, 20.3
ZAPKIN (Chantrapromma *et al.*, 2016 ▸)	4-eth­oxy­phen­yl	4-eth­oxy­phen­yl	17.2	168.4	17.1	13.8

Multiple sets of torsion angles are stated for compounds COGNOD01, DUMWIS, EDUSEE, XOHVOH, MESXEQ, QAJNOG, WIHBUL and XIFTOW because there is more than one independent mol­ecule in their asymmetric units. A third mol­ecule with full mol­ecule disorder in compound WIHBUL was excluded from this table.

**Table 3:** Experimental details

Crystal data	
Chemical formula	C_17_H_12_Br_2_O
*M*_r_	392.09
Crystal system, space group	Monoclinic, *P*2_1_/*c*
Temperature (K)	100
*a*, *b*, *c* (Å)	17.5920 (2), 14.0777 (3), 5.7956 (1)
β (°)	98.742 (1)
*V* (Å^3^)	1418.63 (4)
*Z*	4
Radiation type	Cu *K*α
μ (mm^−1^)	7.17
Crystal size (mm)	0.12 × 0.06 × 0.03

Data collection	
Diffractometer	Agilent SuperNova Dual diffractometer with an Atlas detector
Absorption correction	Multi-scan (*CrysAlis PRO*; Agilent, 2014 ▸)
*T*_min_, *T*_max_	0.64, 0.79
No. of measured, independent and observed [*I* > 2σ(*I*)] reflections	17456, 2524, 2372
*R*_int_	0.031
(sin θ/λ)_max_ (Å^−1^)	0.597

Refinement	
*R*[*F* ^2^ > 2σ(*F* ^2^)], *wR*(*F* ^2^), *S*	0.022, 0.056, 1.15
No. of reflections	2524
No. of parameters	181
H-atom treatment	H-atom parameters constrained
Δρ_max_, Δρ_min_ (e Å^−3^)	0.42, −0.40

Computer programs: *CrysAlis PRO* (Agilent, 2014 ▸), *SHELXS97* (Sheldrick, 2008 ▸), *SHELXL2013* (Sheldrick, 2015 ▸), *Mercury* (Macrae *et al.*, 2006 ▸) and *PLATON* (Spek, 2009 ▸).

## supplementary crystallographic information

### Crystal data

**Table:**

C_17_H_12_Br_2_O	*F*(000) = 768
*M**_r_* = 392.09	*D*_x_ = 1.836 Mg m^−^^3^
Monoclinic, *P*2_1_/*c*	Cu *K*α radiation, λ = 1.54178 Å
*a* = 17.5920 (2) Å	Cell parameters from 11112 reflections
*b* = 14.0777 (3) Å	θ = 4–76°
*c* = 5.7956 (1) Å	µ = 7.17 mm^−^^1^
β = 98.742 (1)°	*T* = 100 K
*V* = 1418.63 (4) Å^3^	Block, yellow
*Z* = 4	0.12 × 0.06 × 0.03 mm

### Data collection

**Table:**

Agilent SuperNova Dual diffractometer with an Atlas detector	2372 reflections with *I* > 2σ(*I*)
Graphite monochromator	*R*_int_ = 0.031
ω scans	θ_max_ = 67.1°, θ_min_ = 4.0°
Absorption correction: multi-scan (CrysAlis PRO; Agilent, 2014)	*h* = −21→18
*T*_min_ = 0.64, *T*_max_ = 0.79	*k* = −16→16
17456 measured reflections	*l* = −6→6
2524 independent reflections	

### Refinement

**Table:**

Refinement on *F*^2^	0 restraints
Least-squares matrix: full	Hydrogen site location: inferred from neighbouring sites
*R*[*F*^2^ > 2σ(*F*^2^)] = 0.022	H-atom parameters constrained
*wR*(*F*^2^) = 0.056	*w* = 1/[σ^2^(*F*_o_^2^) + (0.0179*P*)^2^ + 1.9865*P*] where *P* = (*F*_o_^2^ + 2*F*_c_^2^)/3
*S* = 1.15	(Δ/σ)_max_ = 0.003
2524 reflections	Δρ_max_ = 0.42 e Å^−^^3^
181 parameters	Δρ_min_ = −0.40 e Å^−^^3^

### Special details

**Table:**

Geometry. All esds (except the esd in the dihedral angle between two l.s. planes) are estimated using the full covariance matrix. The cell esds are taken into account individually in the estimation of esds in distances, angles and torsion angles; correlations between esds in cell parameters are only used when they are defined by crystal symmetry. An approximate (isotropic) treatment of cell esds is used for estimating esds involving l.s. planes.

### Fractional atomic coordinates and isotropic or equivalent isotropic displacement parameters (Å^2^)

**Table:**

	*x*	*y*	*z*	*U*_iso_*/*U*_eq_	
Br1	0.14690 (2)	0.38377 (2)	−0.19437 (5)	0.02381 (9)	
Br2	1.00765 (2)	0.35118 (2)	0.21721 (5)	0.02140 (9)	
O1	0.58973 (11)	0.35870 (14)	0.9112 (3)	0.0230 (4)	
C1	0.33281 (15)	0.33927 (19)	0.3232 (4)	0.0190 (5)	
H1A	0.341393	0.310601	0.469408	0.023*	
C2	0.25910 (14)	0.34239 (19)	0.2021 (4)	0.0193 (5)	
H2A	0.218193	0.316997	0.266165	0.023*	
C3	0.24703 (14)	0.38441 (19)	−0.0186 (4)	0.0192 (5)	
C4	0.30666 (15)	0.42466 (18)	−0.1141 (4)	0.0193 (5)	
H4A	0.297492	0.453469	−0.260084	0.023*	
C5	0.38031 (14)	0.42161 (18)	0.0103 (4)	0.0187 (5)	
H5A	0.420665	0.448675	−0.053317	0.022*	
C6	0.39517 (14)	0.37826 (18)	0.2313 (4)	0.0176 (5)	
C7	0.47449 (15)	0.37678 (18)	0.3537 (4)	0.0187 (5)	
H7A	0.512770	0.395031	0.268379	0.022*	
C8	0.49705 (15)	0.35175 (18)	0.5758 (4)	0.0188 (5)	
H8A	0.460021	0.328563	0.659851	0.023*	
C9	0.57702 (15)	0.35856 (18)	0.6962 (4)	0.0194 (5)	
C10	0.64033 (14)	0.36427 (18)	0.5544 (4)	0.0186 (5)	
H10A	0.630488	0.349029	0.396510	0.022*	
C11	0.71104 (14)	0.39078 (18)	0.6484 (4)	0.0169 (5)	
H11A	0.717134	0.413278	0.800928	0.020*	
C12	0.78029 (14)	0.38804 (18)	0.5353 (4)	0.0165 (5)	
C13	0.77978 (14)	0.34717 (18)	0.3144 (4)	0.0166 (5)	
H13A	0.733445	0.327076	0.229449	0.020*	
C14	0.84699 (14)	0.33627 (18)	0.2209 (4)	0.0174 (5)	
H14A	0.846014	0.308769	0.074547	0.021*	
C15	0.91600 (14)	0.36684 (18)	0.3479 (4)	0.0180 (5)	
C16	0.91859 (14)	0.40921 (18)	0.5654 (4)	0.0180 (5)	
H16A	0.964994	0.430278	0.647991	0.022*	
C17	0.85050 (14)	0.41952 (19)	0.6572 (4)	0.0171 (5)	
H17A	0.851669	0.447926	0.802583	0.021*	

### Atomic displacement parameters (Å^2^)

**Table:**

	*U*^11^	*U*^22^	*U*^33^	*U*^12^	*U*^13^	*U*^23^
Br1	0.01735 (14)	0.02515 (16)	0.02724 (15)	0.00374 (11)	−0.00210 (10)	0.00155 (11)
Br2	0.01568 (14)	0.02560 (16)	0.02334 (15)	−0.00101 (10)	0.00433 (10)	−0.00280 (11)
O1	0.0235 (9)	0.0274 (11)	0.0181 (9)	−0.0039 (8)	0.0034 (7)	0.0004 (7)
C1	0.0212 (13)	0.0189 (13)	0.0173 (12)	0.0008 (10)	0.0041 (10)	−0.0004 (10)
C2	0.0175 (12)	0.0185 (13)	0.0225 (13)	−0.0010 (10)	0.0054 (10)	0.0002 (10)
C3	0.0182 (12)	0.0179 (13)	0.0209 (12)	0.0038 (10)	0.0009 (10)	−0.0009 (10)
C4	0.0252 (13)	0.0153 (13)	0.0174 (12)	0.0039 (11)	0.0040 (10)	−0.0002 (10)
C5	0.0203 (12)	0.0163 (13)	0.0208 (12)	−0.0001 (10)	0.0074 (10)	−0.0009 (10)
C6	0.0200 (13)	0.0141 (12)	0.0192 (12)	0.0006 (10)	0.0048 (10)	−0.0035 (10)
C7	0.0183 (12)	0.0165 (13)	0.0225 (12)	−0.0013 (10)	0.0067 (10)	−0.0013 (10)
C8	0.0183 (12)	0.0169 (13)	0.0220 (13)	−0.0023 (10)	0.0054 (10)	−0.0026 (10)
C9	0.0218 (13)	0.0157 (13)	0.0213 (13)	0.0002 (10)	0.0050 (10)	−0.0005 (10)
C10	0.0194 (13)	0.0193 (14)	0.0168 (12)	0.0009 (10)	0.0022 (10)	−0.0008 (10)
C11	0.0190 (12)	0.0144 (13)	0.0171 (11)	0.0020 (10)	0.0022 (9)	0.0001 (9)
C12	0.0170 (12)	0.0147 (12)	0.0175 (12)	0.0007 (10)	0.0014 (9)	0.0031 (10)
C13	0.0151 (12)	0.0175 (13)	0.0157 (11)	−0.0007 (10)	−0.0022 (9)	0.0009 (9)
C14	0.0201 (12)	0.0166 (13)	0.0147 (11)	0.0000 (10)	0.0005 (10)	−0.0002 (10)
C15	0.0177 (12)	0.0179 (13)	0.0187 (12)	0.0021 (10)	0.0034 (10)	0.0034 (10)
C16	0.0166 (12)	0.0188 (13)	0.0172 (12)	−0.0013 (10)	−0.0019 (9)	0.0005 (10)
C17	0.0195 (12)	0.0173 (13)	0.0137 (11)	−0.0015 (10)	−0.0007 (9)	0.0003 (10)

### Geometric parameters (Å, º)

**Table:**

Br1—C3	1.896 (3)	C8—H8A	0.9300
Br2—C15	1.895 (2)	C9—C10	1.484 (4)
O1—C9	1.232 (3)	C10—C11	1.333 (4)
C1—C2	1.378 (4)	C10—H10A	0.9300
C1—C6	1.402 (4)	C11—C12	1.469 (3)
C1—H1A	0.9300	C11—H11A	0.9300
C2—C3	1.396 (4)	C12—C17	1.399 (3)
C2—H2A	0.9300	C12—C13	1.402 (3)
C3—C4	1.380 (4)	C13—C14	1.382 (4)
C4—C5	1.384 (4)	C13—H13A	0.9300
C4—H4A	0.9300	C14—C15	1.389 (4)
C5—C6	1.407 (4)	C14—H14A	0.9300
C5—H5A	0.9300	C15—C16	1.389 (4)
C6—C7	1.466 (4)	C16—C17	1.390 (4)
C7—C8	1.336 (4)	C16—H16A	0.9300
C7—H7A	0.9300	C17—H17A	0.9300
C8—C9	1.475 (4)		
			
C2—C1—C6	121.6 (2)	C8—C9—C10	118.9 (2)
C2—C1—H1A	119.2	C11—C10—C9	121.4 (2)
C6—C1—H1A	119.2	C11—C10—H10A	119.3
C1—C2—C3	118.7 (2)	C9—C10—H10A	119.3
C1—C2—H2A	120.6	C10—C11—C12	126.6 (2)
C3—C2—H2A	120.6	C10—C11—H11A	116.7
C4—C3—C2	121.5 (2)	C12—C11—H11A	116.7
C4—C3—Br1	119.17 (19)	C17—C12—C13	118.3 (2)
C2—C3—Br1	119.33 (19)	C17—C12—C11	119.7 (2)
C3—C4—C5	119.1 (2)	C13—C12—C11	121.8 (2)
C3—C4—H4A	120.4	C14—C13—C12	121.1 (2)
C5—C4—H4A	120.4	C14—C13—H13A	119.5
C4—C5—C6	121.2 (2)	C12—C13—H13A	119.5
C4—C5—H5A	119.4	C13—C14—C15	119.3 (2)
C6—C5—H5A	119.4	C13—C14—H14A	120.4
C1—C6—C5	117.9 (2)	C15—C14—H14A	120.4
C1—C6—C7	123.6 (2)	C16—C15—C14	121.3 (2)
C5—C6—C7	118.5 (2)	C16—C15—Br2	119.99 (19)
C8—C7—C6	126.3 (2)	C14—C15—Br2	118.71 (19)
C8—C7—H7A	116.9	C15—C16—C17	118.7 (2)
C6—C7—H7A	116.9	C15—C16—H16A	120.6
C7—C8—C9	124.1 (2)	C17—C16—H16A	120.6
C7—C8—H8A	117.9	C16—C17—C12	121.3 (2)
C9—C8—H8A	117.9	C16—C17—H17A	119.3
O1—C9—C8	119.5 (2)	C12—C17—H17A	119.3
O1—C9—C10	121.6 (2)		
			
C6—C1—C2—C3	0.9 (4)	O1—C9—C10—C11	−15.2 (4)
C1—C2—C3—C4	−1.7 (4)	C8—C9—C10—C11	165.4 (2)
C1—C2—C3—Br1	176.9 (2)	C9—C10—C11—C12	171.9 (2)
C2—C3—C4—C5	1.2 (4)	C10—C11—C12—C17	179.3 (3)
Br1—C3—C4—C5	−177.34 (19)	C10—C11—C12—C13	−6.2 (4)
C3—C4—C5—C6	0.0 (4)	C17—C12—C13—C14	1.4 (4)
C2—C1—C6—C5	0.3 (4)	C11—C12—C13—C14	−173.2 (2)
C2—C1—C6—C7	179.9 (2)	C12—C13—C14—C15	−0.3 (4)
C4—C5—C6—C1	−0.8 (4)	C13—C14—C15—C16	−0.8 (4)
C4—C5—C6—C7	179.6 (2)	C13—C14—C15—Br2	179.91 (19)
C1—C6—C7—C8	−10.2 (4)	C14—C15—C16—C17	0.9 (4)
C5—C6—C7—C8	169.4 (3)	Br2—C15—C16—C17	−179.82 (19)
C6—C7—C8—C9	−175.0 (2)	C15—C16—C17—C12	0.1 (4)
C7—C8—C9—O1	160.7 (3)	C13—C12—C17—C16	−1.3 (4)
C7—C8—C9—C10	−19.8 (4)	C11—C12—C17—C16	173.4 (2)

### Hydrogen-bond geometry (Å, º)

*Cg*1 and *Cg*2 are the centroids of the C1–C16 and C12–C17 rings, respectively.

**Table:**

*D*—H···*A*	*D*—H	H···*A*	*D*···*A*	*D*—H···*A*
C1—H1*A*···*Cg*1^i^	0.93	2.86	3.539 (3)	131
C14—H14*A*···*Cg*2^ii^	0.93	2.74	3.428 (3)	131

Symmetry codes: (i) *x*, −*y*+1/2, *z*+1/2; (ii) *x*, −*y*+1/2, *z*−1/2.
